# Fecal Metabolites Were Altered, Identified as Biomarkers and Correlated With Disease Activity in Patients With Systemic Lupus Erythematosus in a GC-MS-Based Metabolomics Study

**DOI:** 10.3389/fimmu.2020.02138

**Published:** 2020-09-10

**Authors:** Ren Yan, Huiyong Jiang, Silan Gu, Ninghan Feng, Nan Zhang, Longxian Lv, Fengping Liu

**Affiliations:** ^1^State Key Laboratory for Diagnosis and Treatment of Infectious Diseases, National Clinical Research Center for Infectious Diseases, Collaborative Innovation Center for Diagnosis and Treatment of Infectious Diseases, The First Affiliated Hospital, College of Medicine, Zhejiang University, Hangzhou, China; ^2^Department of Urology, Affiliated Wuxi No.2 People's Hospital, Nanjing Medical University, Wuxi, China; ^3^Wuxi School of Medicine, Jiangnan University, Wuxi, China

**Keywords:** metabolomics, autoimmune disease, biomarkers, systemic lupus erythematosus, GC-MS

## Abstract

Gut metabolites are products of the crosstalk between microbes and their host and play an important role in the occurrence, development, diagnosis, and treatment of autoimmune diseases. This work profiled the fecal metabolome of patients with systemic lupus erythematosus (SLE) using gas chromatography–mass spectrometry (GC-MS) and analyzed the potential roles of metabolites in the diagnosis and development of SLE. Fecal sample from 29 SLE patients without any other diseases and 30 healthy controls (HCs) were analyzed by metabolomics profiling. All participants took no antibiotics in the month before sampling and clinical data collecting. The metabolome profiles of patients with SLE and HCs were significantly different. Thirty fecal metabolites, such as deoxycholic acid, erucamide, L-tryptophan and putrescine, were significantly enriched, while nine metabolites, such as glyceric acid, γ-tocopherol, (Z)-13-octadecenoic acid and 2,4-di-tert-butylphenol, were depleted in SLE patients vs. HCs. The areas under the curve (AUCs) of L-valine, pyrimidine, erucamide, and L-leucine during ROC analysis were 0.886, 0.833, 0.829, and 0.803, indicating their good diagnostic potential. Moreover, the combination of L-valine, erucamide and 2,4-di-tert-butylphenol gave an AUC of 0.959. SLE-altered metabolites were significantly located in 28 pathways, such as ABC transporters (*p* = 3.40E-13) and aminoacyl-tRNA biosynthesis (*p* = 2.11E-12). Furthermore, SLE-altered fecal metabolites were closely correlated with SLE indicators, e.g., L-tryptophan was positively correlated with the SLEDAI-2K (*p* = 0.007). Our results suggest that the SLE fecal metabolome is closely associated with the occurrence and development of SLE and is of great diagnostic value.

## Introduction

Systemic lupus erythematosus (SLE) is a chronic autoimmune disease. Tissue damage mediated by patient autoantibodies can cause multiple organ failure and endanger life. The etiology and pathogenesis of SLE are unclear. Its clinical symptoms are complex and changeable, which makes SLE easily confused with other autoimmune diseases. Currently, the diagnosis of SLE is based on a series of criteria issued by the American College of Rheumatology (ACR) (1, 2). For SLE to be diagnosed, four of the eleven ACR criteria must be met. This diagnostic method based on typical clinical manifestations often leads to delayed or missed diagnoses. Due to the lack of rapid and effective diagnostic criteria, the interval between the first symptoms and diagnosis of SLE averaged 21.82 months (3), resulting in irreversible organ damage, reduction in life quality, and even death. Although other indicators, such as anti-nuclear, anti-dsDNA, anti-ssDNA, anti-nucleosome antibodies and anti-cellular antibody, are also used as auxiliary indicators for SLE diagnosis (4, 5), these biomarkers can also be positive in other autoimmune diseases and healthy controls (HCs).

Recently, some potential SLE biomarkers have been identified among DNA, RNA, proteins, and some small molecules (6, 7). The metabolome can reflect cellular processes resulting from the genome, transcriptome, and proteome, thus reflecting physiological and pathological changes. For example, the levels of glutamate, 2-hydroxyisobutyrate and citrate in serum and valine, leucine, and 3-hydroxyisobutyrate in urine were significantly different between SLE patients and HCs (8, 9). Furthermore, during the occurrence and development of diseases, an in-depth understanding of pathogenesis is very important to analyse the alterations in metabolic networks before and after stimulation or interference (such as gene mutations and environmental changes) (10). For instance, concentrations of sorbitol, fructose, and lactate increased significantly in the cerebrospinal fluid of multiple sclerosis patients, suggesting that mitochondrial dysfunction is involved in the pathogenesis of multiple sclerosis progression (11).

In this work, we analyzed the metabolome of the feces of SLE patients and HCs using gas chromatography–mass spectrometry (GC-MS) to identify potential diagnostic biomarkers among fecal metabolites and to disclose the potential correlations of fecal metabolites with SLE development and disease activity.

## Methods

### Study Population

The participants consisted of 29 patients with SLE and 30 gender- and age-matched HCs. Diagnosis of SLE was made according to the ACR criteria (2). None of the participants took antibiotics 1 month before sampling. The protocol of this study was approved by the ethics committee of Zhejiang University. All participants signed informed consent prior to enrolment. Fresh blood samples were used to assay routine blood indicators, complement proteins, liver functions, and renal functions, such as the levels of leukocytes, lymphocytes, monocytes, erythrocyte sedimentation rate (ESR), total protein, alanine transaminase (ALT), aspartate aminotransferase (AST), alkaline phosphatase (ALP), creatinine, uric acid, and urea nitrogen. Disease activity was assessed with the SLE Disease Activity Index 2000 (SLEDAI-2K) score (12).

### Preparation of Fecal Samples for GC Analysis

Fecal samples were pre-treated as we described previously for GC-MS analysis (13). Briefly, 15 mg of thawed feces sample was mixed with 800 μL of ice-cold methanol for extraction; the mixture was homogenized and centrifuged, and then the supernatant was filtered through a 0.22-μm membrane filter. After 20 μL of heptadecanoic acid (1 mg/mL) was added as an internal standard, the filtered supernatant was vacuum freeze-dried, methoxymated, and trimethylsilylated.

### Metabolomics Profiling

The pre-treated samples were analyzed on an Agilent 7890A-5975C GC-MS system (Agilent, USA). All samples were running singly, a quality control (QC) sample made by mixing and blending equal volumes (10 μL) of each fecal sample was used. The raw data obtained from GC-MS runs were analyzed using Agilent Qualitative Analysis version B.07.00 software. Metabolites were identified against the NIST 17 database with a matching score of at least 80. The resulting dataset was normalized to the internal standard before multivariate analysis. Orthogonal partial least squares discriminant analysis (OPLS-DA) was performed to visualize metabolic differences between two groups using the SIMCA version 14.1 software from Umetrics. Metabolic pathway analysis was performed using the online software MetaboAnalyst 4.0 (https://www.metaboanalyst.ca) (14) based on the Kyoto Encyclopedia of Genes and Genomes (KEGG).

### Statistical Analysis

The Mann-Whitney U test was used to compare any two data sets that were not normally distributed; otherwise, one-way ANOVA followed by the Student-Newman-Keuls method was used. The clinical data of SLE patients and HCs were expressed as the mean ± standard error or the median with the 25th and 75th percentiles. Spearman's rank correlation test was used to analyse the correlations. Differential metabolites were selected according to variable importance in the projection (VIP) values obtained from the OPLS-DA model and the *p*-values from the Mann-Whitney *U*-test or one-way ANOVA; metabolites with VIP values >1.0 and *p* < 0.05 were included.

## Results

### Characteristics of SLE Patients and Healthy Controls

Twenty-nine SLE patients who had no other major diseases and had not used antibiotics within a month participated in the study (Table 1 and Supplemental Table S1). Their average age was ~45 years, and ~90% of them were women. Thirty age- and gender-matched HCs were recruited as well. The median duration of illness in SLE patients was ~6.50 years. The median SLEDAI-2K score of SLE activity was 7.00. The SLE of 31% of patients was inactive, 51.7% was mild, 10.4% was moderate, and 6.9% was severe. Compared with those of HCs, serum levels of neutrophils and monocytes increased in SLE patients, while levels of lymphocyte, erythrocyte, hemoglobin, haematocrit, mean corpuscular hemoglobin concentration, total protein, albumin, total bilirubin, and indirect bilirubin decreased (Table 1).

**Table 1:** Demographic and clinical features of patients with systemic lupus erythematosus and healthy controls.

	**SLE (*n* = 29)**	**HCs (*n* = 30)**	***p*-value**
Age (years)^a^	44.76 ± 2.80	44.80 ± 2.09	9.90E-01
Male/female	3/26	3/27	–
Illness duration (years)^b^	6.50 (2.75, 11.00)	–	–
Arthritis (%)	9 (31.03)	–	–
Ulceration (%)	4 (13.79)	–	–
Rash (%)	5 (17.24)	–	–
Proteinuria (%)	3 (10.34)	–	–
Edema (%)	9 (31.03)	–	–
Complement 3 (g/L)^b^	0.72 (0.5, 0.81)	–	–
Complement 4 (g/L)^b^	0.16 (0.11, 0.20)	–	–
IgG (g/L)^b^	14.20 (11.38, 17.93)	–	–
IgA (g/L)^b^	2.29 (1.48, 2.95)	–	–
IgM (g/L)^b^	0.90 (0.70, 1.62)	–	–
C-reactive protein (mg/L)^b^	3.11 (1.82, 27.88)	–	–
Erythrocyte sedimentation rate (mm/h)^b^	28.50 (10.50, 50.00)	–	–
SLEDAI-2K^b^	7.00(4.00, 9.00)	–	–
Prednisone (mg/day)^b^	30 (20, 50)	–	–
Hydroxychloroquine (g/day)^b^	0.40 (0.20, 0.40)	–	–
Neutrophil (%)^a^	72.32 ± 2.43	59.61 ± 1.63	4.00E-05
Lymphocyte (%)^a^	18.24 ± 2.10	32.98 ± 1.43	2.25E-07
Monocyte (%)^a^	8.02 ± 0.74	5.59 ± 0.36	2.07E-03
Lymphocyte (10^9^/L)^a^	1.03 ± 0.13	1.83 ± 0.08	6.03E-07
Monocyte (10^9^/L)^a^	0.48 ± 0.05	0.31 ± 0.02	2.19E-03
Erythrocyte (10^12^/L)^a^	3.92 ± 0.12	4.74 ± 0.08	4.39E-07
Hemoglobin (g/L)^a^	111.52 ± 5.28	138.20 ± 2.24	7.57E-05
Haematocrit (L/L)^a^	0.35 ± 0.01	0.42 ± 0.01	8.30E-05
Mean corpuscular hemoglobin concentration (g/L)^a^	318.81 ± 4.31	331.80 ± 1.62	9.12E-03
Total protein (g/L)^a^	68.40 ± 2.17	74.48 ± 0.73	1.36E-02
Albumin (g/L)^a^	38.55 ± 1.50	47.18 ± 0.46	1.17E-05
Total bilirubin (mg/dL)^b^	0.47 (0.32, 0.68)	0.64 (0.47, 0.83)	4.70E-02
Indirect bilirubin (mg/dL)^b^	0.30 (0.19, 0.44)	0.47 (0.29, 0.60)	1.10E-02

a *Mean ± standard error*.

b *Median (25th and 75th percentile)*.

*SLE, systemic lupus erythematosus; HCs, healthy controls; IgG, immunoglobulin G; SLEDAI-2K, SLE disease activity index 2000*.

### Fecal Metabolome Profiles Distinguish SLE Patients From HCs

**Figure 1:**
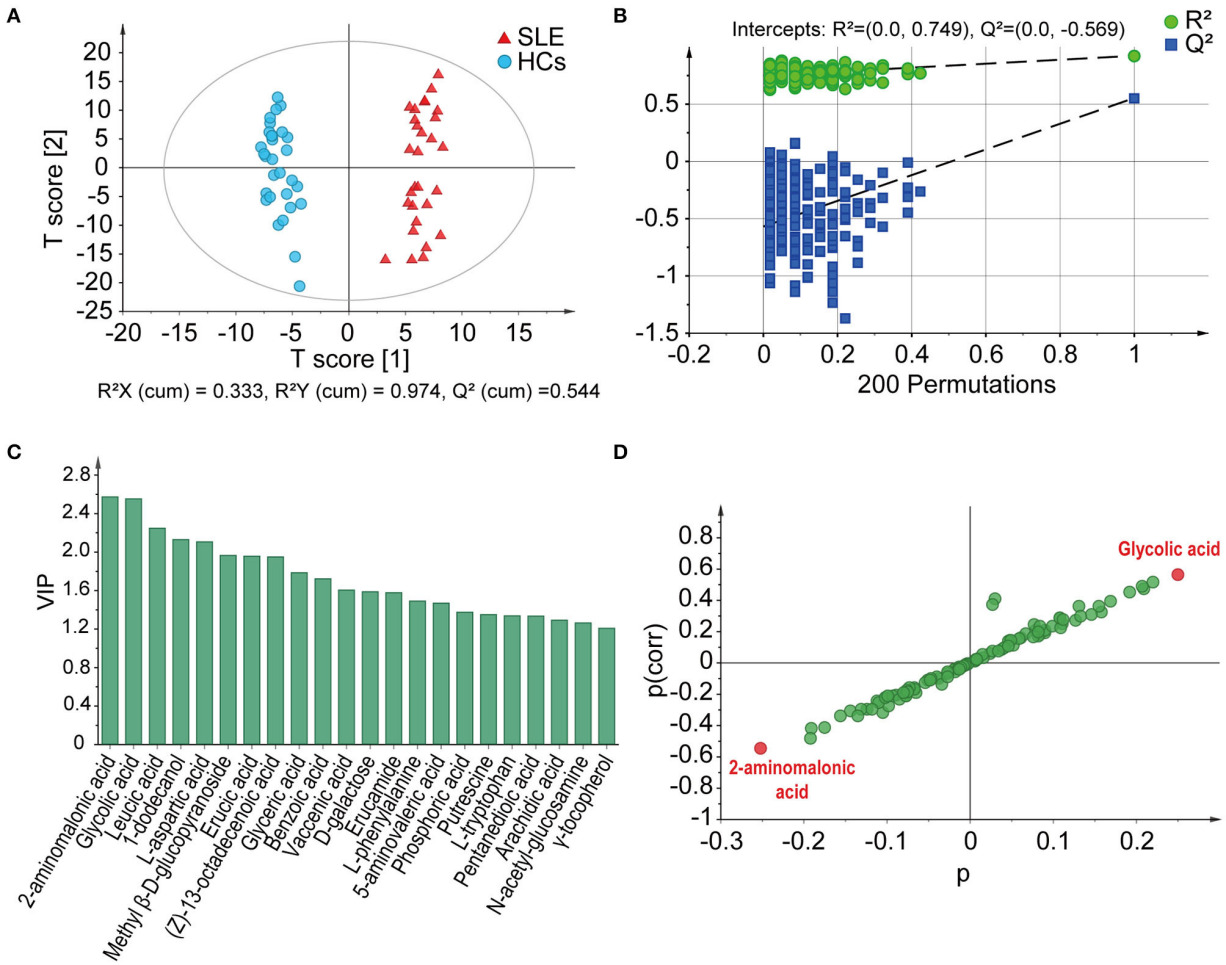
GC-MS-based fecal metabolome profiles distinguish SLE patients from HCs. **(A)** Score plots based on OPLS-DA illustrated that the metabolic profiles of SLE patients and HCs were clearly separated. **(B)** Statistical validation of the OPLS-DA model by permutation test. **(C)** The VIP values of those metabolites have important contributions to distinguishing between SLE patients and HCs. **(D)** 2-Aminoalonic acid and glycolic acid were selected as potential biomarkers based on the S-plot. SLE, systemic lupus erythematosus; HCs, healthy controls; OPLS-DA, orthogonal partial least squares discriminant analysis; VIP, variable importance in the projection.

In GC-MS-based metabolomics analyses, we identified 105 metabolites pertaining to 14 groups, including fatty acids, amino acids, cholic acids, nucleotides, amines, alcohols, and monosaccharides, from the feces of all participants. In the plot results of OPLS-DA model, the SLE patients and HCs were clearly separated into two different clusters [R^2^X (cum) = 0.333, R^2^Y (cum) = 0.974, Q^2^ (cum) = 0.544] (Figure 1A), showing that there were different metabolic profiles between these two groups. The permutation test (*n* = 200) was performed to validate the reliability of the prediction model. The slope of the straight line was large, and the intercept of *Q*^2^ was −0.569, indicating that the OPLS-DA model did not overfit (Figure 1B). The VIP of the OPLS-DA model is a quantitative statistical parameter ranking metabolites according to their ability to discriminate between two groups. Twenty two metabolites were found to have VIP values >1.0, indicating their important contributions in distinguishing between SLE patients and HCs (Figure 1C). In descending order, they were 2-aminomalonic acid, glycolic acid, leucic acid, 1-dodecanol, L-aspartic acid, methyl β-D-glucopyranoside, erucic acid, (Z)-13-octadecenoic acid, glyceric acid, benzoic acid, vaccenic acid, D-galactose, erucamide, L-phenylalanine, 5-aminovaleric acid, phosphoric acid, putrescine, L-tryptophan, pentanedioic acid, arachidic acid, N-acetyl-glucosamine, and γ-tocopherol. On the basis of its position in the S-plot, we identified 2-aminomalonic acid and glycolic acid as potential biomarkers for distinguishing these two groups (Figure 1D).

### The Distributions of Many Metabolites in SLE and HCs Feces Were Significantly Different

**Figure 2:**
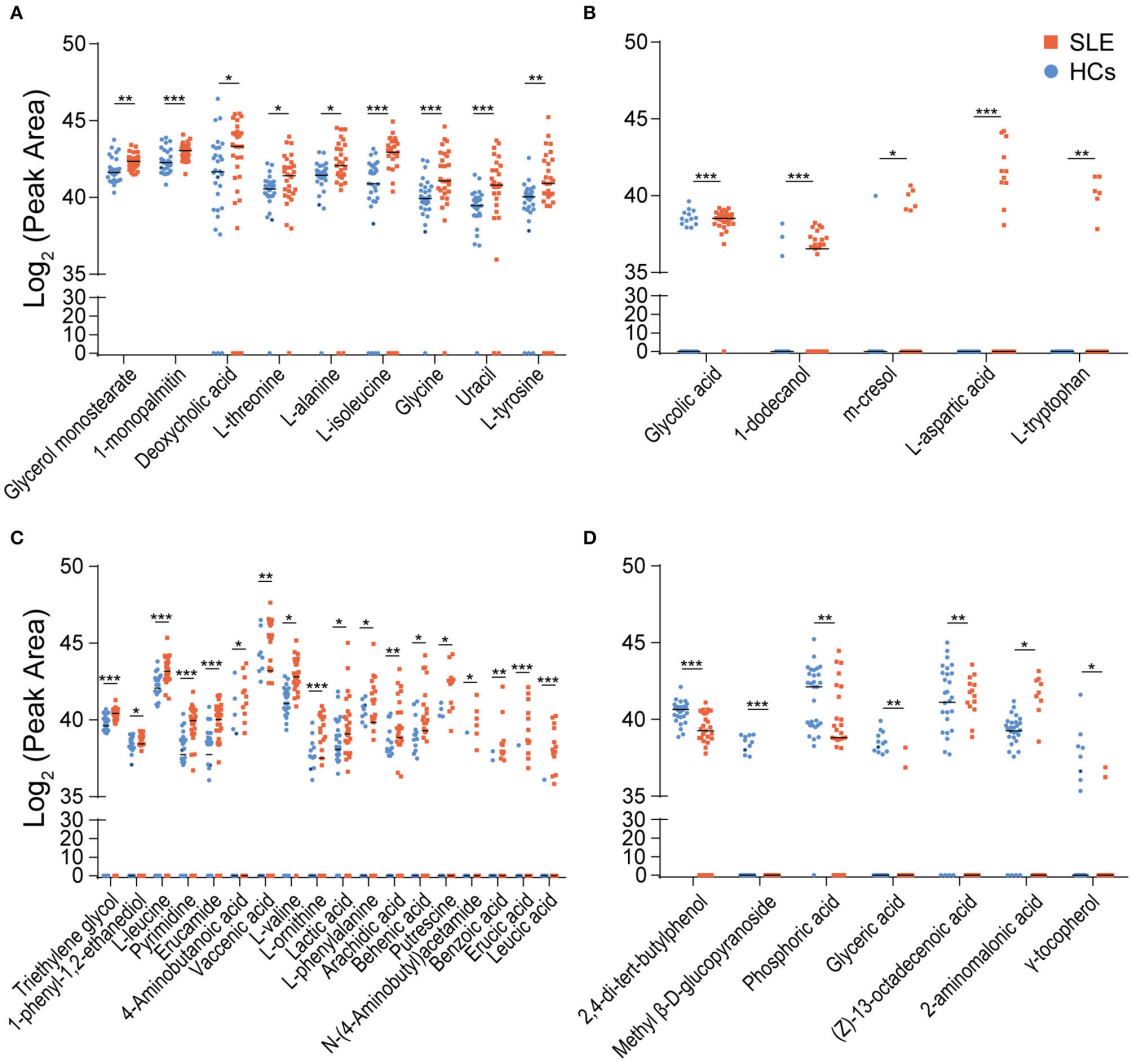
Distribution of differential metabolites in SLE and HCs feces. **(A)** Nine metabolites were enriched in SLE feces but had similar detection rates in the HCs and SLE groups. **(B)** Five metabolites had increased detection rates in SLE feces. Among them, L-aspartic acid and L-tryptophan were detected in only SLE feces **(C)**. Eighteen metabolites were enriched and had higher detection rates in SLE feces than in HCs feces. **(D)** Seven metabolites were depleted and had lower detection rates in SLE feces than in HCs feces (*P < 0.05; **P < 0.01; and ***P < 0.001). SLE, systemic lupus erythematosus; HCs, healthy controls.

Using parametric or non-parametric analyses, we found that there were significant differences in the distribution of 39 metabolites in the feces of SLE and HCs individuals (*p* < 0.05). Among them, the VIP value of 18 metabolites were >1.0. Whereas, D-galactose (VIP = 1.59), 5-aminovaleric acid (VIP = 1.47), pentanedioic acid (VIP = 1.34), and N-acetyl- glucosamine (VIP = 1.27) were found to be insignificant. First, 30 metabolites were enriched in SLE feces. Compared with those in HCs feces, the detection rates of glycerol monostearate, 1-monopalmitin, deoxycholic acid, L-threonine, L-alanine, L-isoleucine, glycine, uracil, and L-tyrosine were almost unchanged, but their peak areas were significantly larger in SLE feces (Figure 2A). Glycolic acid, 1-dodecanol, and m-cresol had increased detection rates in SLE feces, but the average peak area of the detected samples was similar in HCs and SLE feces. L-aspartic acid and L-tryptophan were detected in only SLE feces (Figure 2B). Triethylene glycol, 1-phenyl-1,2-ethanediol, L-leucine, pyrimidine, erucamide, 4-aminobutanoic acid, vaccenic acid, L-valine, L-ornithine, lactic acid, L-phenylalanine, arachidic acid, behenic acid, putrescine, N-(4-aminobutyl)acetamide, benzoic acid, erucic acid, and leucic acid had both higher peak areas and higher detection rates in SLE feces than in HCs feces (Figure 2C). Second, 7 metabolites were depleted in SLE feces. In addition, 2,4-di-tert-butylphenol, phosphoric acid, glyceric acid, (Z)-13-octadecenoic acid and γ-tocopherol decreased in both their detection rates and average peak areas in SLE feces compared with HCs feces. Methyl β-D-glucopyranoside was detected in only HCs feces. The detection rate of 2-amino malonic acid in SLE was greatly reduced, although its average peak area in the SLE samples was higher than that in the HCs samples (Figure 2D).

### Several Fecal Metabolites Exhibit Good Diagnostic Potential for SLE

**Figure 3:**
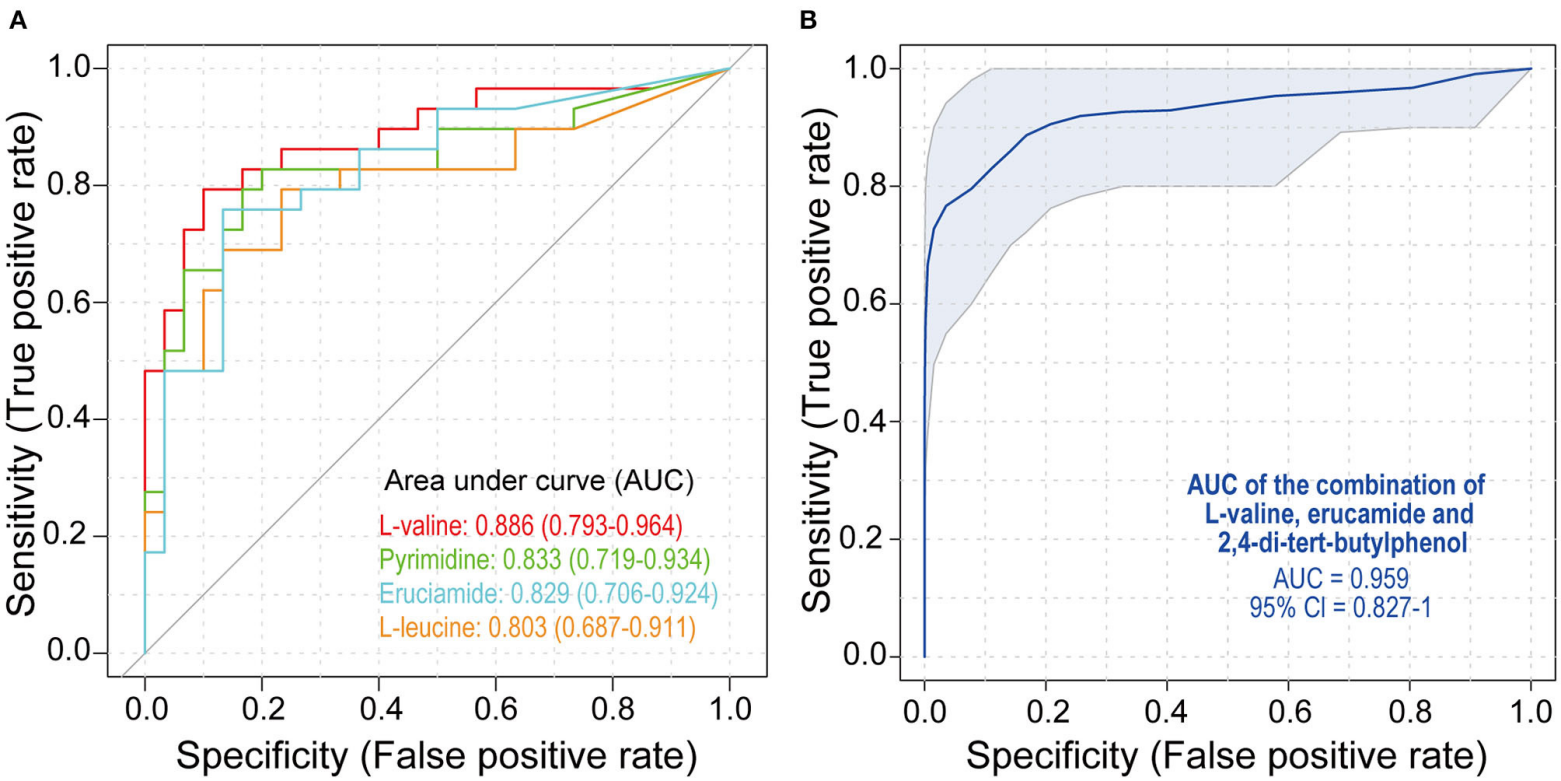
Receiver operating characteristic (ROC) analysis of metabolites. **(A)** ROC curves of L-valine, pyrimidine, erucamide, and L-leucine. **(B)** ROC curves of the combination of L-valine, erucamide, and 2,4-di-tert-butylphenol. SLE, systemic lupus erythematosus; HCs, healthy controls; ROC, receiver operating characteristic.

To study the diagnostic capacity of continuous markers, receiver operating characteristic (ROC) curve analysis was conducted. The area under the curve (AUC) of four metabolites, L-valine (AUC = 0.886), pyrimidine (AUC = 0.833), erucamide (AUC = 0.829), and L-leucine (AUC = 0.803), was between 0.80 and 0.90, indicating their good diagnostic performance (Figure 3A). The combination of L-valine, erucamide and 2,4-di-tert-butylphenol exhibited increased diagnostic potential (AUC = 0.959) (Figure 3B).

### SLE-Altered Metabolites Were Significantly Located in 28 Pathways

**Figure 4:**
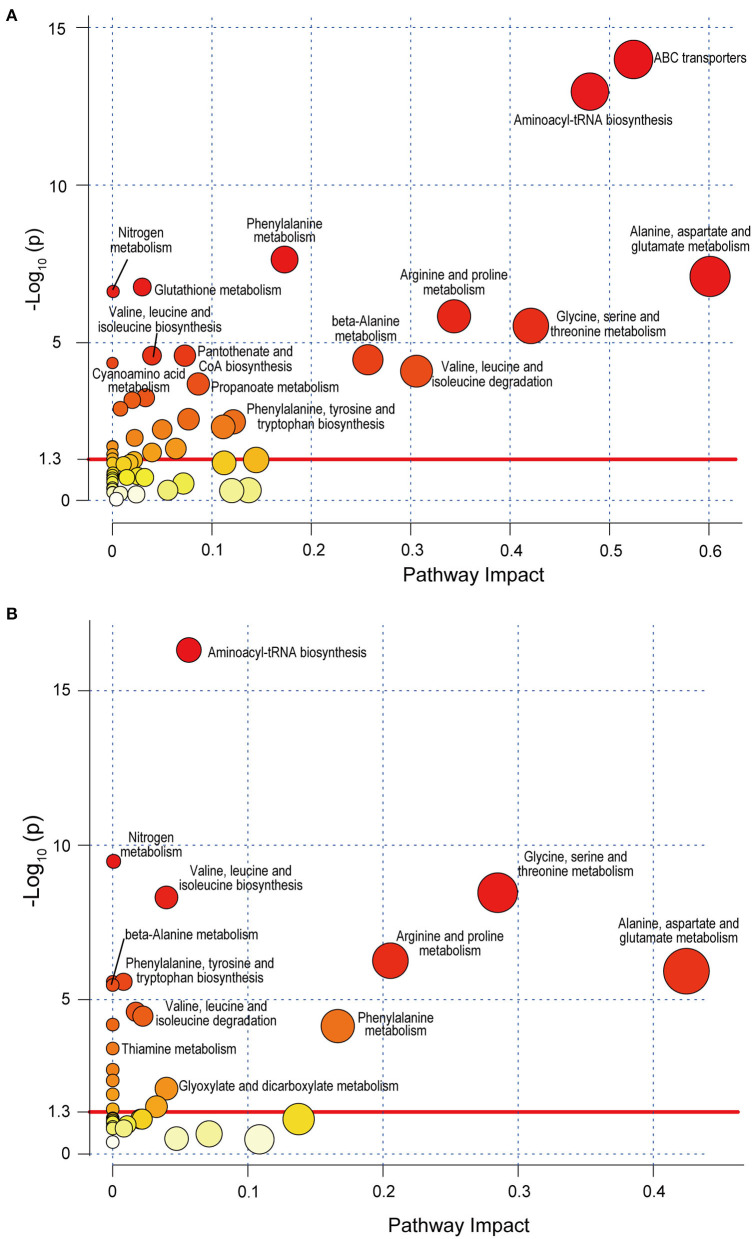
Metabolic pathway analysis of SLE-altered metabolites using MetaboAnalyst 4.0 based on the KEGG. Circle colors indicate pathway enrichment significance. Circle size indicates the extent of pathway impact. Circle above the red line (–Log_10_ *P* > 1.30) represents significantly enriched pathway. **(A)** Pathway enrichment analysis based on KEGG full library. **(B)** Pathway enrichment analysis based on KEGG *Homo sapiens* library. SLE, systemic lupus erythematosus; KEGG, Kyoto Encyclopedia of Genes and Genomes.

Of the 39 SLE-altered metabolites submitted to the KEGG, 29 metabolites were encoded in the KEGG, and 25 metabolites were matched to 28 metabolic pathways. Among all these pathways, ABC transporters (*p* = 3.40E-13) and aminoacyl-tRNA biosynthesis (*p* = 2.11E-12) were the most significant (Figure 4A). First, 10 pathways were related to amino acids. Among them, there were six amino acid metabolic pathways: glycine, serine and threonine metabolism; arginine, and proline metabolism; alanine, aspartate, and glutamate metabolism; β-alanine metabolism; glutathione metabolism; and phenylalanine metabolism. Two amino acid synthesis pathways were present: valine, leucine, and isoleucine biosynthesis and phenylalanine, tyrosine and tryptophan biosynthesis. The other two amino acid pathways were valine, leucine, and isoleucine degradation and aminoacyl-tRNA biosynthesis. Second, three alkaloid synthesis pathways were significant: biosynthesis of alkaloids derived from ornithine, lysine and nicotinic acid; tropane, piperidine, and pyridine alkaloid biosynthesis; and biosynthesis of alkaloids derived from the shikimate pathway. Third, five other metabolic pathways were significant, and they included cyanoamino acid metabolism, thiamine metabolism, nitrogen metabolism, and propanoate metabolism. Fourth, four other synthetic pathways were significant: pantothenate and CoA biosynthesis, biosynthesis of secondary metabolites, biosynthesis of unsaturated fatty acids, and biosynthesis of plant hormones. In addition, other important pathways included ABC transporters, glucosinolate biosynthesis, Parkinson's disease, carbon fixation in photosynthetic organisms, the two-component system, and the yeast cell cycle.

When only the human database (*Homo sapiens* library) was selected, 8 of the above 28 metabolic pathways were not found. They were the pathways biosynthesis of alkaloids derived from ornithine, lysine, and nicotinic acid; biosynthesis of alkaloids derived from the shikimate pathway; biosynthesis of plant hormones; carbon fixation in photosynthetic organisms; cell cycle—yeast; glutathione metabolism; tropane, piperidine, and pyridine alkaloid biosynthesis; and two-component systems (Figure 4B). Moreover, the enrichment of ABC transporters pathway (*p* = 0.027) changed greatly when using *Homo sapiens* library for analysis. This suggests that some SLE-altered fecal metabolites are possibly located in the pathways of organisms other than humans, such as microorganisms.

### SLE-Altered Fecal Metabolites Were Closely Correlated With SLE Indicators

**Figure 5:**
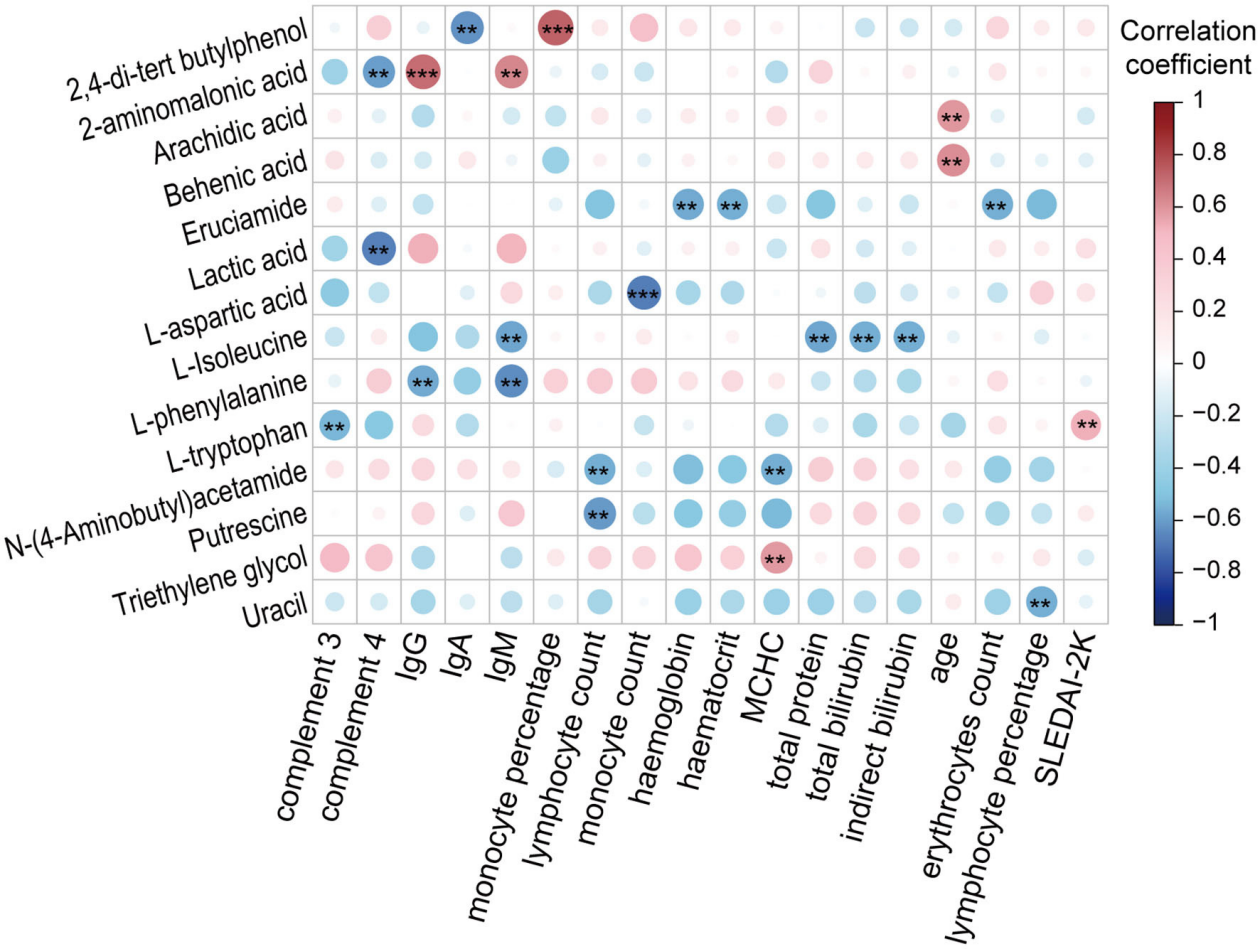
Associations between SLE-altered metabolites and clinical SLE indicators (**P < 0.01 and ***P < 0.001). IgG, immunoglobulin G; IgA, immunoglobulin A; IgM, immunoglobulin M; MCHC, mean corpuscular hemoglobin concentration; SLEDAI-2K, SLE disease activity index 2000.

To gain insight into the potential relationship between fecal metabolites and SLE, we performed a Spearman correlation analysis (Figure 5 and Supplemental Figure S1). The correlations between 39 SLE-altered metabolites and 49 clinical parameters, such as age, BMI, drug treatment, disease activity score, and serum indicators (e.g., ESR, CRP, Lymphocyte count), were tested. As a result, a total of 119 significant correlations (*p* < 0.05) were found between 35 metabolites and 42 clinical parameters. When using the absolute value of correlation coefficient *r* > 0.4 and *p* < 0.01 as a screening threshold, the detection of L-tryptophan in only SLE feces was positively correlated with the disease activity index SLEDAI-2K (*r* = 0.52, *p* = 0.007), suggesting an important potential role for fecal L-tryptophan in affecting and determining SLE activity. Second, enrichment in long-chain fatty acids, arachidic acid, and behenic acid was positively correlated with age, which may be a reflection of the deterioration of fatty acid absorption capacity with aging. Third, some serum immune indicators were negatively correlated with 10 metabolites and positively correlated with 2 metabolites. These negative correlations include 2-aminomalonic acid and lactic acid with complement 4, L-tryptophan with complement 3, N-(4-aminobutyl)acetamide, and putrescine with lymphocyte count, uracil with lymphocyte percentage, L-aspartic acid with monocyte count, L-isoleucine with IgM, 2,4-di-tert-butylphenol with IgA, and L-phenylalanine with IgG and IgM. In contrast, there were positive correlations of 2-aminomalonic acid with IgG or IgM and of 2,4-di-tert-butylphenol with monocyte percentage. Fourth, some parameters related to erythrocytes were correlated with three metabolites. There were negative correlations for erucamide with the level of erythrocytes, haematocrit, or hemoglobin and mean erythrocyte hemoglobin with N-(4-aminobutyl)acetamide. Mean erythrocyte hemoglobin was positively correlated with triethylene glycol. In addition, L-isoleucine was negatively correlated with liver function indicators, such as total bilirubin, indirect bilirubin, and total protein.

## Discussion

The role of metabolism in the development and diagnosis of autoimmune diseases is receiving increasing attention. Our results showed that there were significant differences in the fecal metabolome between SLE patients and HCs during GC-MS analysis. Thirty-nine metabolites differently distributed in these two groups and were located in 28 pathways. Five metabolites, such as L-valine, were of great value in the diagnosis of SLE. L-tryptophan was positively correlated with the disease activity index of SLE. These results have implications for the diagnosis and treatment of SLE.

Both our results based on GC-MS and those previously reported based on LC-MS indicate unique fecal metabolites. Among our 39 SLE-altered fecal metabolites, only tyrosine was also observed to be enriched, and the remaining 38 metabolites were not reported in the LC-MS study. Similarly, 23 of the 24 SLE-altered fecal metabolites detected by LC-MS analysis were not found in our results (15). In the LC-MS-based analysis, proline, and methionine were enriched in SLE patients compared with HCs. Although they were also identified in our research, they did not differentially distribute between the two groups. Moreover, 21 SLE-altered metabolites in LC-MS analysis were not identified in our results based on GC-MS. Among these metabolites, asparagine, D,L-pipecolinic acid, glycyl-L-proline, carnosine, xanthurenic acid, kynurenic acid, 1,2-dioleoyl-rac-glycerol, monoacylglycerol 22:6, monoacylglycerol 16:5, lysophosphatidylethanolamine 16:0, lysophosphatidylcholine 22:5, and phosphatidylglycerol 27:2 were enriched in SLE feces compared with HCs feces, whereas D-ala-D-ala, lauryl diethanolamide, sulfoquinovosyl diacylglycerol, adenosine, adenosine 5′-diphosphate, trigonelline, thiamine pyrophosphate, and mucic acid were depleted (15). The difference in the results obtained by the GC-MS and LC-MS methods is mainly due to the differing intrinsic natures of these methods, in particular, their sensitivity or separation and/or extraction efficiency. Although some compounds were detected by both methods, each method had its own unique compounds. This phenomenon is prevalent in many studies using the same sample and different metabolome analysis methods. For example, in a metabolome study using the same urine sample, the compounds detected and identified by GC-MS and LC-MS varied widely (16). Therefore, all results from different metabolic methods are meaningful, even though these cross-study comparisons are limited by differences in samples, technologies, and bioinformatics.

Our results showed that amino acids, fatty acids, and their related metabolites were enriched in SLE feces compared to HCs feces. On the one hand, the fecal enrichment of amino acids corresponds to plasma depletion of amino acids in SLE compared to HCs that was observed in other studies. We detected 16 amino acids, 11 of which were enriched in SLE feces, and 5 were not significantly different between HCs and SLE feces. Meanwhile, some compounds related to amino acid metabolism, such as 2-aminomalonic acid, 4-aminobutanoic acid, putrescine, N-(4-aminobutal)acetamide, and benzoic acid, were also enriched in SLE feces. Yan et al. found that 15 amino acids were reduced in SLE plasma compared to HCs plasma, including the SLE-enriched fecal L-threonine, L-alanine, L-leucine, glycine, L-valine, L-tyrosine, L-phenylalanine, L-tryptophan and aminomalonate in this study (9). Ouyang et al. found that L-alanine, L-isoleucine, L-lysine, L-phenylalanine, L-tyrosine, and L-valine were depleted in the plasma of SLE patients (17). In addition, several studies have also found that the plasma amino acids were depleted in SLE patients compared with HCs (18, 19). On the other hand, fatty acids and their derivatives were enriched in SLE feces compared to HCs feces. Several studies have reported that fatty acids in SLE plasma were generally depleted compared to those in HCs, although fatty acids altered in feces and plasma do not have a strict one-to-one correspondence. For example, we detected glycolic acid, vaccenic acid, lactic acid, arachidic acid, behenic acid, erucic acid, and leucic acid enrichment in SLE feces, while the depletion of many fatty acids, such as vaccenic acid, lactic acid, malic acid and fumaric acid, was reported in several other studies (20). Therefore, we hypothesize that in patients with SLE, the fecal enrichment and plasma depletion of amino acids and fatty acids and the fecal enrichment of deoxycholic acid, glycerol monostearate, and 1-monopalmitin may be related to intestinal damage but do not exclude the contribution of possibly accelerated degradation of these compounds in patients (21). At least 50% of SLE patients have gastrointestinal symptoms, including nausea, vomiting, anorexia, abdominal pain, diarrhea, and abdominal distension (22). Notably, six essential amino acids, L-valine, L-leucine, L-isoleucine, L-phenylalanine, L-threonine, and L-tryptophan, were listed in the metabolites depleted in plasma but enriched in feces. They have important physiological functions, such as regulation of immunity, metabolism and neural activity. Therefore, the causal relationship and mechanism of their alterations are worthy of further study.

Our results showed that the SLE-enriched fecal amino acids were significantly located in ABC transporters and aminoacyl-tRNA biosynthesis pathways. Amino acids are mainly absorbed in the intestinal mucosal cells of humans through the carrier protein and γ-glutamine cycle. Intestinal prokaryotes and archaea acquire amino acids from the environment primarily through type I ABC transporters in the third subgroup (23). These amino acids can be used for microbial protein synthesis involving aminoacyl-tRNA biosynthesis, microbial energy metabolism, and conversion into a variety of physiologically active substances, such as neurotransmitters and hormones. Therefore, the amino acids enriched in feces have important influences on the physiological activities of both microorganisms and their host. For example, SLE-enriched fecal branched-chain amino acids, including L-leucine, L-isoleucine, and L-valine, can serve as both precursors for membrane fatty acids and key coregulators for the growth and virulence of pathogenic bacteria. A low-proline or low-protein diet in germ-free mice colonized by a dysbiotic human gut microbiota resulted in reduced expansion of wild-type *Clostridioides difficile* after challenge, suggesting that amino acid availability might be important for *C. difficile* infection (24). Tryptophan is important for the growth of some pathogens, and severe tryptophan deficiency prevents the regular onset of *Chlamydia trachomatis* and reduces its reactivation (25). These may also explain why the enrichment of ABC transporters pathway is quite different when the full library and *Homo sapiens* library are used for analysis. Thus, the contribution of SLE-enriched fecal amino acids to disease is worthy of further study.

Our results showed that some SLE-altered metabolites were closely correlated with immunity. First, three essential amino acids, tryptophan, isoleucine, and phenylalanine, which are also three of the four glucogenic and ketogenic amino acids, were negatively correlated with at least one of complement 3, IgG, IgM, total protein, or total bilirubin; the excitatory amino acid aspartate was negatively correlated with monocyte counts. Other studies have reported that these amino acids can regulate the above-mentioned immune and hepatic functional factors, although their results may not be uniform due to differences in models. For example, phenylalanine is a potential player in modulating innate immunity and adaptive immunity and can promote the transcription of C3b when injected into zebrafish (26). Our results showed that alterations in amino acids were opposite between feces and those found in blood previously (9). The negative correlations of fecal amino acids with immune factors were consistent with the conclusion in several studies that deletion of blood amino acids destroys immunity (27–29). Furthermore, SLE-enriched fecal tryptophan, isoleucine, phenylalanine, and aspartic acid were the main metabolites of our study and contributed to glucosinolates, alkaloids, plant hormones, and other pathways of intestinal microbes, which in turn affected host health. Second, 2-aminomalonic acid is not only a genotoxic microbial-derived compound but also an essential unit of precolibactin, which is a precursor of the carcinogenic compound colibactin produced by microbes such as *Escherichia coli* (30). The high positive correlation of 2-aminomalonic acid with serum IgG and IgM most likely reflected the interaction of its producer and host. The compound 2,4-di-tert-butylphenol, which has a wide range of food and microbial sources, has antibacterial and antioxidative activity. Its depletion may result from reduced sources and/or increased oxidative stress in SLE feces (31). Its negative correlation with IgA and positive correlation with monocyte percentage indicates that microbes play an important role in their production. Interestingly, SLE-enriched fecal erucamide, which was negatively correlated with erythrocytes, hemoglobin and haematocrit, is potently proangiogenic, and activates angiogenin (32). Thus, some SLE-enriched fecal metabolites have potential health hazards.

Our results showed that fecal metabolites have potential diagnostic value. On the one hand, some metabolites may contribute to the prompt and correct diagnosis of SLE. The manifestations of SLE are varied, including symptoms in the gastrointestinal tract, skin, kidney, blood, nerves, and other organs. Even in the same organ or tissue, the symptoms may be different. Some patient symptoms change with time. Therefore, the diagnosis of SLE is very challenging (33). It was reported that the combination of PG 27:2 and proline (AUC = 0.846) in feces (15), kynurenine (AUC = 0.859) in peripheral blood lymphocytes (34), as well as methionine (AUC = 0764) in serum (9) achieved good predictive ability of SLE. Our results found that the combination of fecal L-valine, erucamide, and 2,4-di-tert-butylphenol predicted SLE well (AUC = 0.959). On the other hand, metabolites play important roles in predicting the activity of SLE. Our results showed that fecal L-tryptophan was positively correlated with the SLEDAI-2K score (*r* = 0.52, *p* = 0.007). Serum-depleted tryptophan is a discriminatory metabolite of SLE (18). Furthermore, decreased levels of tryptophan could also be connected to the kynurenine pathway, which is related to the activated immune response and many disorders, including SLE (18). The disease activity score of SLEDAI or SLEDAI-2K was previously reported to be associated with the N-acetylaspartate/creatine ratio (*r* = −0.41) in insula (35), the docosapentaenoic acid (*r* = 0.321) and docosahexaenoic acid (*r* = 0.339) in plasma (36), as well as the hexose-phosphate (*r* = 0.492) and other metabolites in peripheral blood lymphocytes (34). Because some of the first symptoms of SLE are in the intestine, the use of intestinal metabolites is likely to become an important means of early warning and predicting the occurrence and development of SLE.

In conclusion, the identification of different metabolites in SLE and HCs feces is of great significance to the pathogenesis, diagnosis, and treatment of SLE diseases and to metabolomic studies on blood and urine. Integrating the metabolic results for different body parts and techniques and conducting large-scale validation will be some of the important aspects of SLE basic and clinical research in the future.

## Data Availability Statement

All datasets generated for this study are included in the article/Supplementary Material.

## Ethics Statement

The studies involving human participants were reviewed and approved by the ethics committee of Zhejiang University. The patients/participants provided their written informed consent to participate in this study.

## Author Contributions

RY, LL, and FL conceived and designed the study. RY, HJ, and SG performed the experiments and analyzed the data. NF and NZ contributed to the sample collection and storage. NF and FL were responsible for clinical data collection. RY and LL wrote the manuscript. All authors contributed to the article and approved the submitted version.

## Conflict of Interest

The authors declare that the research was conducted in the absence of any commercial or financial relationships that could be construed as a potential conflict of interest.
