# Supplementary material for: Fecal Metabolites Were Altered, Identified as Biomarkers and Correlated With Disease Activity in Patients With Systemic Lupus Erythematosus in a GC-MS-Based Metabolomics Study
Source: Front Immunol. 2020 Sep 10;11:2138. doi: 10.3389/fimmu.2020.02138 (PMC7511511; doi:10.3389/fimmu.2020.02138)
Supplement: Supplementary file 2 [file Data_Sheet_1.docx]

**Supplementary materials**

**Supplemental Table S1** Detailed clinical features of patients with systemic lupus erythematosus.

Please see the excel document.

**Supplemental Figure S1** Associations between SLE-altered metabolites and clinical SLE indicators (0.05 < P < 0.01).

Abbreviations: Body mass index, BMI; Alanine transaminase, ALT; Aspartate aminotransferase, AST; Immunoglobulin G, IgG, Immunoglobulin M, IgM, Erythrocyte sedimentation rate ESR
