# Supplementary figures and images for: Fecal Metabolites Were Altered, Identified as Biomarkers and Correlated With Disease Activity in Patients With Systemic Lupus Erythematosus in a GC-MS-Based Metabolomics Study
Source: Front Immunol. 2020 Sep 10;11:2138. doi: 10.3389/fimmu.2020.02138 (PMC7511511; doi:10.3389/fimmu.2020.02138)

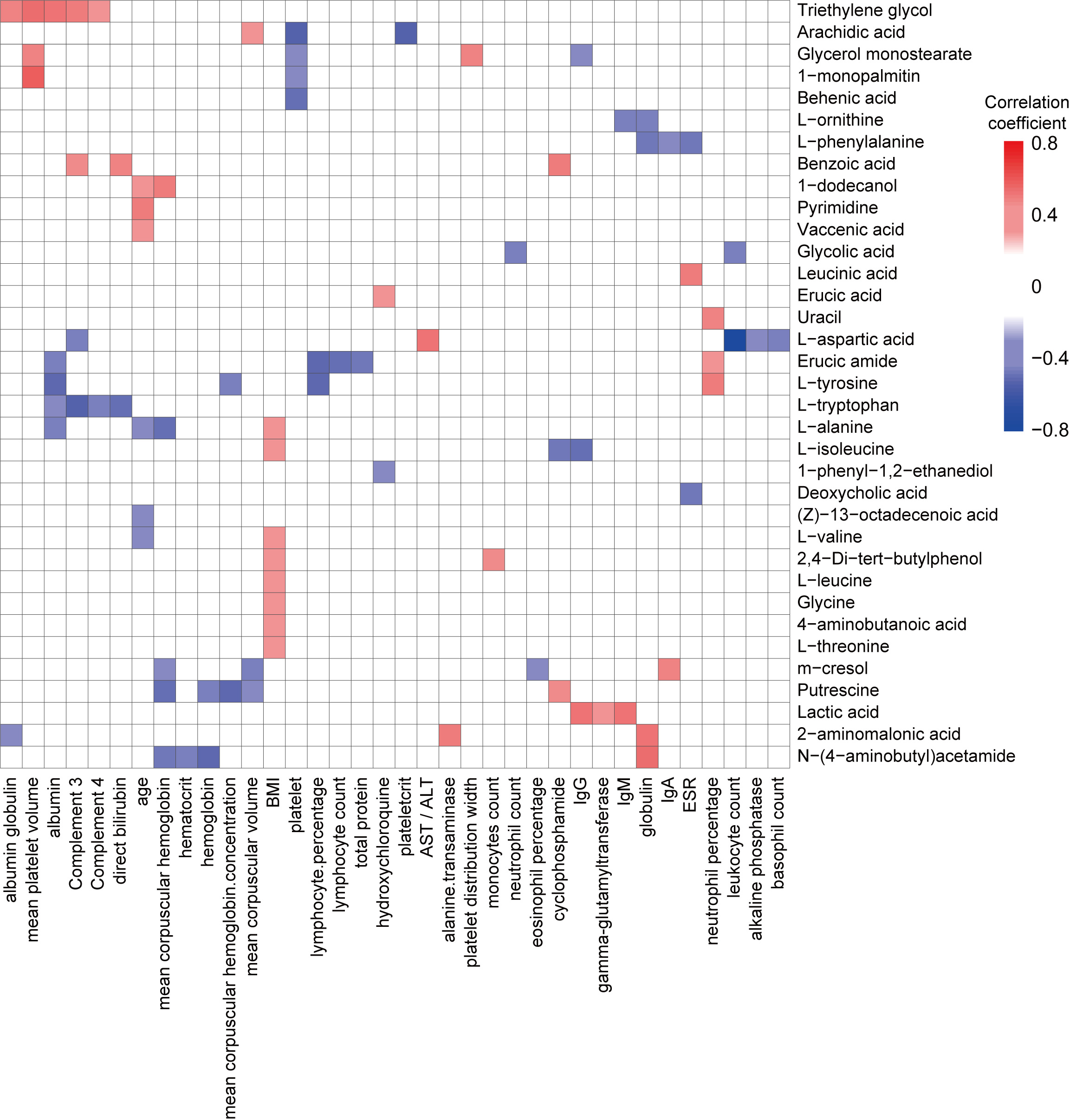

Supplement: Supplementary file 3 [file Image_1.TIF]
